# Successful retrograde transvenous embolization under balloon occlusion for rectal arteriovenous malformation

**DOI:** 10.1007/s12328-020-01335-w

**Published:** 2021-01-08

**Authors:** Amane Jubashi, Daisuke Yamaguchi, Goshi Nagatsuma, Suma Inoue, Yuichiro Tanaka, Wataru Yoshioka, Naoyuki Hino, Tomohito Morisaki, Keisuke Ario, Kenichiro Fukui, Hideki Ishimaru, Seiji Tsunada

**Affiliations:** 1 Department of Gastroenterology, National Hospital Organization Ureshino Medical Center, Ureshino, Japan; 2 Division of Gastroenterology, Department of Internal Medicine, Saga University, Saga, 849-8501 Japan; 3 Department of Radiology, National Hospital Organization Ureshino Medical Center, Ureshino, Japan; 4 Division of Radiology, Nagasaki University, Nagasaki, Japan

**Keywords:** Arteriovenous malformation, Interventional radiology, Transcatheter embolization

## Abstract

A 57-year-old man was admitted to our hospital because of frequent hematochezia. Colonoscopy exhibited a submucosal tumor-like lesion in the lower rectum. Abdominal contrast-enhanced computed tomography showed a rectal arteriovenous malformation (AVM) on the right side wall of the lower rectum. The feeder was the superior rectal artery, with early venous return. Embolization of the draining vein and feeding artery of the AVM with N-butyl-2-cyanoacrylate under balloon occlusion was planned. Angiography of the superior rectal artery showed the nidus in the rectum with early venous return of contrast material. The portal vein was punctured percutaneously under ultrasound guidance, and a balloon catheter advanced to the distal part of the superior rectal vein. Venography under balloon occlusion showed the outflow vein and nidus. Transvenous and transarterial nidus embolization with N-butyl-2-cyanoacrylate under balloon occlusion was then performed. Since the embolization, there have been no further episodes of bleeding. There is no established treatment for AVMs. Successful treatment requires targeting and eradication of the nidus. We successfully performed embolization therapy for a rectal AVM via a retrograde transvenous approach. This technique may be suitable for completely eradicating the nidus without the risk of embolism.

## Introduction

Intestinal arteriovenous malformations (AVMs) are a common cause of lower gastrointestinal bleeding [1, 2]. AVMs have generally been treated surgically, either by ligation of the afferent arteries or by attempts at excision. Less invasive treatments such as endoscopic treatment and transcatheter arterial embolization (TAE) have been reported; however, additional treatment may be required for recurrence after treatment with these modalities [3, 4]. We report here a case of successful embolization therapy for a rectal AVM via a balloon-occluded retrograde transvenous approach.

## Case report

A 57-year-old man was admitted to our hospital because of painless hematochezia after defecation. He had facial and conjunctival pallor consistent with anemia. His medical history included a coronary intervention for myocardial infarction, and he was taking two antiplatelet drugs. Blood tests showed a mild microcytic and hypochromic anemia.

**Fig. 1:**
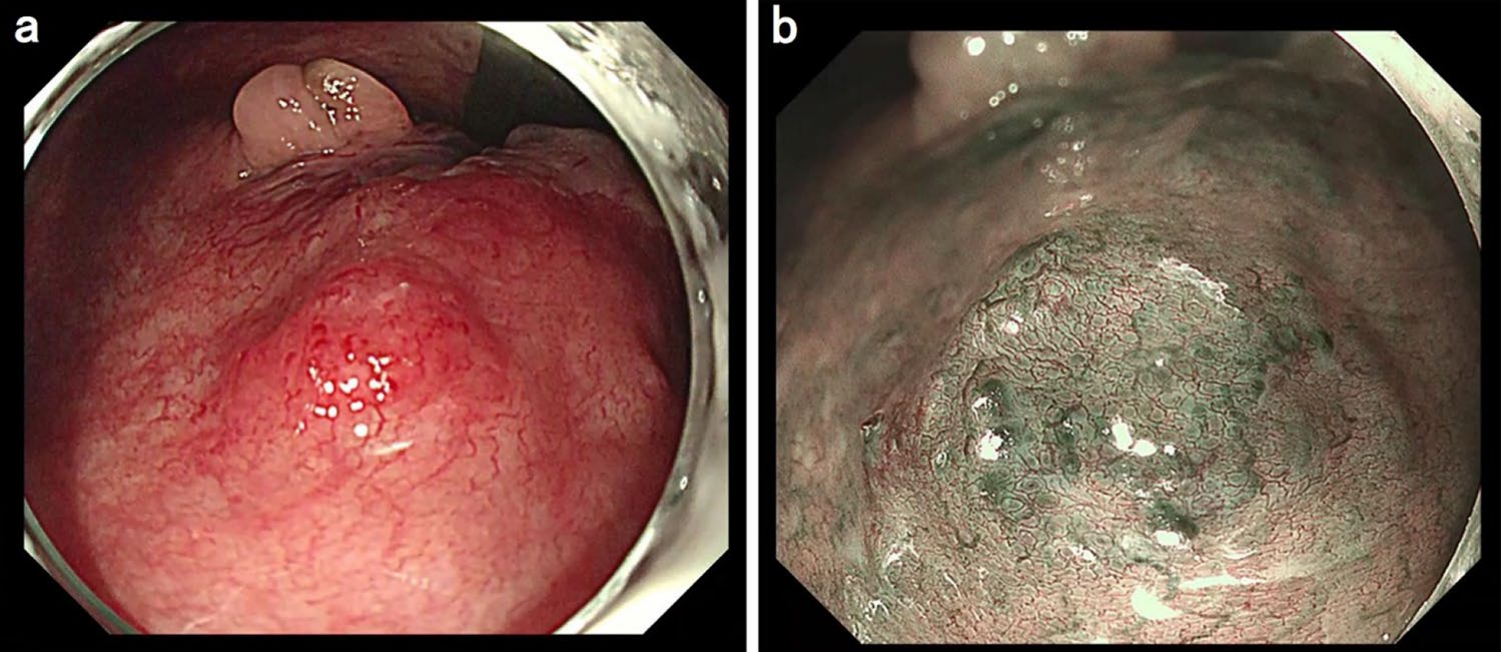
Colonoscopy images showing **a** a pulsating,10 mm diameter, submucosal tumor-like lesion in the lower rectum and **b** tortuous and enlarged blood vessels are visible on the surface of the mucous membrane

**Fig. 2:**
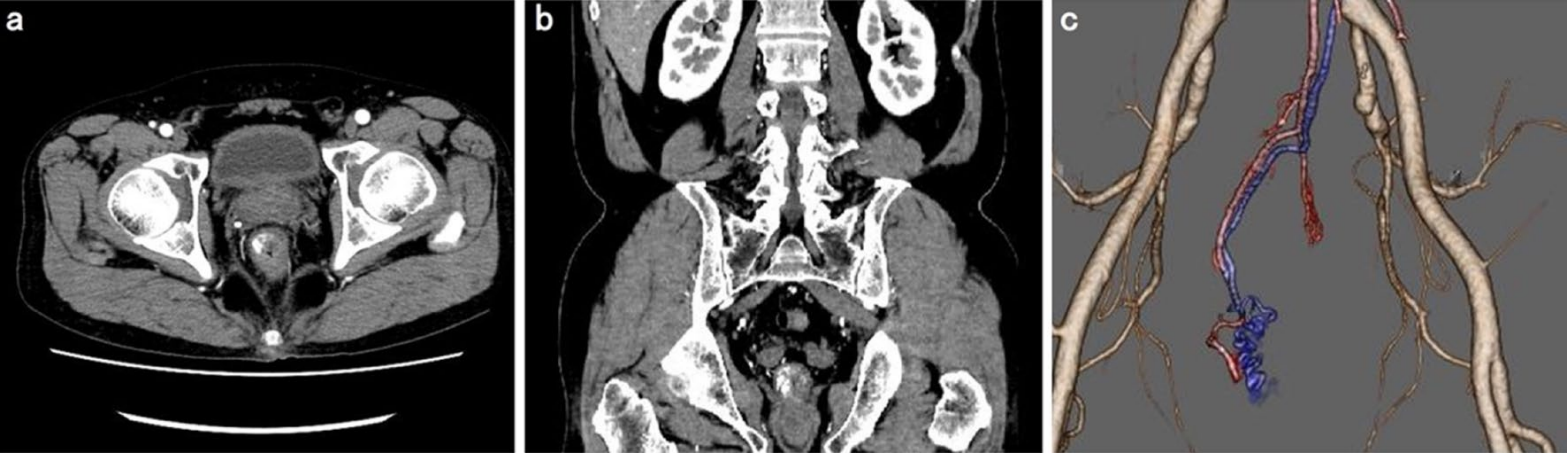
**a**, **b** CECT images showing a cluster of abnormal vessels in the lower rectum. **c** 3D-reconstruction of CECT findings revealing that the superior rectal artery is supplying blood flow to the lesion

**Fig. 3:**
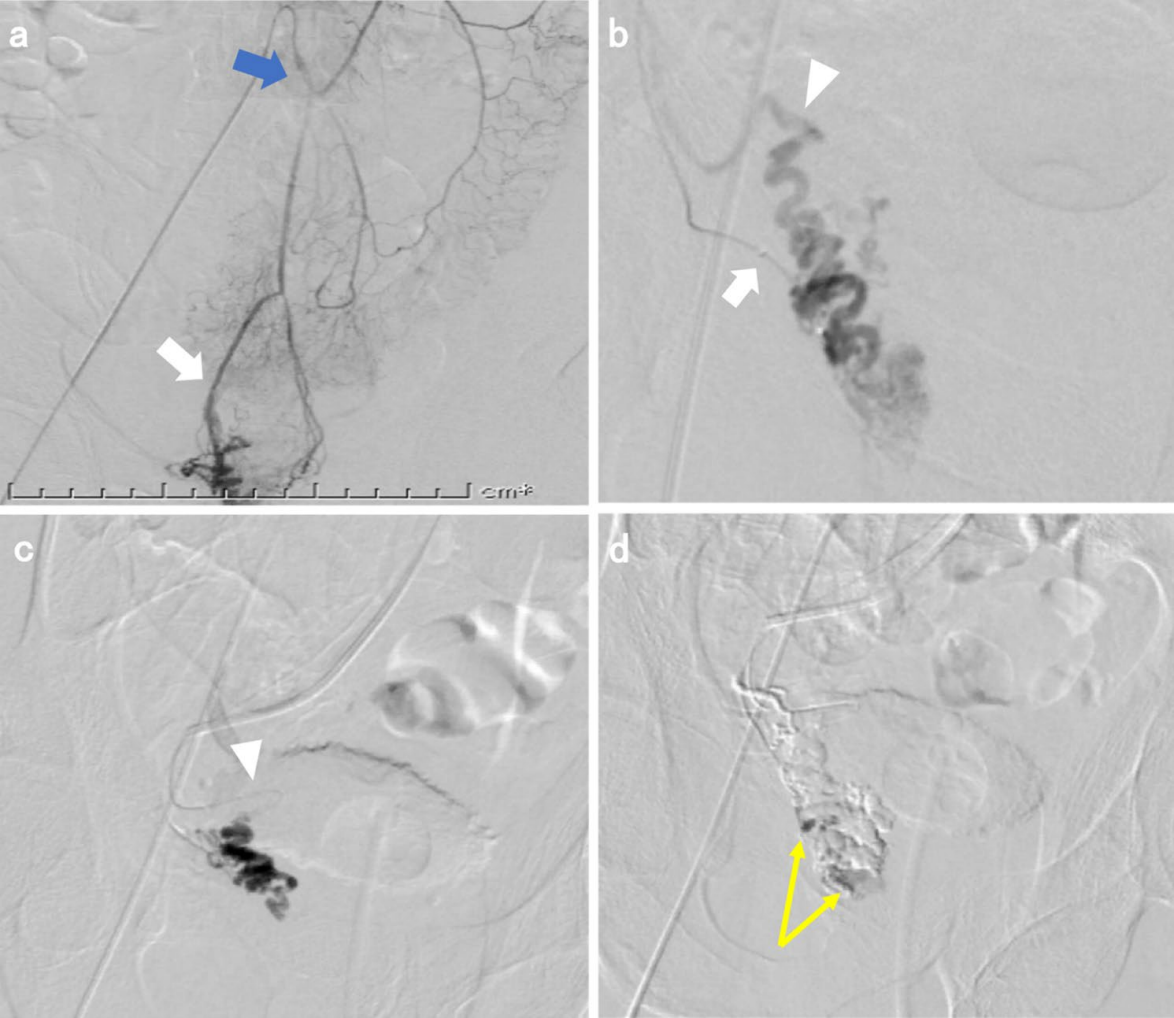
**a** Angiography via the IMA (blue arrow, upper panel). SRA were visualized (white arrow, under panel). **b** Angiography via the SRA (white arrow, lower panel). The nidus and an outflow vein, SRV (arrowhead, upper panel) were visualized. **c** Venography via the SRV (arrowhead) under balloon occlusion showed outflow vein and nidus; 20% NBCA–lipiodol was retrogradely injected intravenously to embolize the nidus. **d** Angiography via the SRA after embolization. Only a vestige of the vascular malformation was detected (yellow arrow); additional arterial embolization was therefore performed

**Fig. 4:**
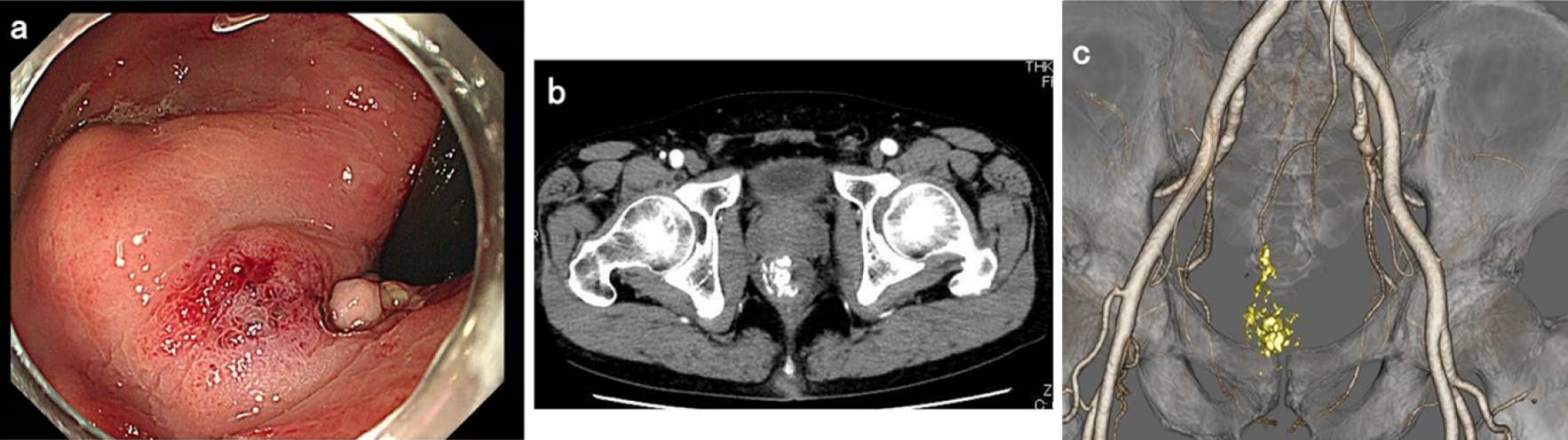
**a** Colonoscopy images obtained one month after discharge showing a ridge but no pulsation. **b**, **c** CECT and 3D-reconstruction of the CECT reveals adequate lipiodol accumulation and resolution of the premature perfusion of the rectal vein in the arterial phase

Colonoscopy revealed a pulsating submucosal tumor-like lesion in the lower rectum (Fig. 1a, b). Tortuous enlarged blood vessels were visible on surface of the mucous membrane. The lesion was suspected to be the cause of bleeding; however, no active bleeding was observed during colonoscopy. An abdominal contrast-enhanced computed tomography (CECT) showed a collection of dilated blood vessels on the right side wall of the lower rectum. The feeder was the superior rectal artery (SRA), with early venous return (Fig. 2a–c). Given that hemostasis had occurred spontaneously, we decided to perform elective angiography and embolization. During the hospitalization, he had a further episode of hemorrhage and spontaneous hemostasis and required a blood transfusion for his anemia. After discontinuation of the antiplatelet drugs and heparinization, angiography was performed for the purpose of diagnosis and treatment on the seventh day of hospitalization. Angiography of the SRA showed vascular hyperplasia and nidus (Fig. 3a, b), resulting in a definitive diagnosis of a rectal AVM. A percutaneous transhepatic portal vein puncture was performed under ultrasound guidance and a catheter advanced to the distal part of the superior rectal vein (SRV). Venography under balloon occlusion showed the outflow vein and nidus with stasis of blood flow. To achieve an adequate embolic effect, the balloon was dilated upstream of the inflowing artery to reduce arterial blood flow, after which 20% NBCA–lipiodol (1:4) was slowly and retrogradely injected intravenously to embolize the nidus (Fig. 3c). Because a remaining vestige of the vascular malformation was detected by SRA after the retrograde transvenous obliteration procedure, TAE under balloon dilatation in the SRA was performed with 20% NBCA–lipiodol (Fig. 3d). Angiography performed via the inferior mesenteric artery (IMA) confirmed that the nidus had disappeared and the early venous return resolved. Following embolization of this patient’s rectal AVM, his bleeding on defecation stopped completely, his symptoms of anemia improved, and his hemoglobin concentration returned to within the normal range. He was discharged on the 11th postoperative day without any adverse events or rebleeding. Lower gastrointestinal endoscopy and CECT performed one month after discharge revealed submucosal tumor-like ridges but no pulsation, sufficient lipiodol accumulation in the region that the AVM had been in, and resolution of the early return of the rectal vein in the arterial phase disappeared (Fig. 4a–c). Six months after the embolization, no further bleeding had occurred.

## Discussion

An AVM is an abnormal connection between arteries and veins that bypasses the capillary system. Histopathologically, AVMs in the gastrointestinal tract are characterized by diffuse vasodilation in all layers from the submucosal to the serosal layer and are composed of an inflow artery (feeder), an abnormal blood vessel assembly (nidus), and an outflow vein (drainer) [5, 6]. Patients typically present with intermittent bloody stools without abdominal pain, and anemia; the differential diagnosis is chronic hemorrhage from diverticulae. The lesions are most often located in the right hemicolon (37%), followed by the jejunum (24%), and ileum (19%), rectal lesions being rare (8%) [4].

AVMs are generally treated by surgical excision of the nidus. However, being highly invasive, this procedure may be contraindicated in older patients who are in poor general condition. In addition, if the lesion is located in the rectum, as in the present case, a permanent colostomy is required, prejudicing the patient’s quality of life. One alternative approach to small lesions is endoscopic hemostasis with clips and coagulation [4]. However, achieving hemostasis by this means is reportedly difficult and, even when it has been achieved, and the recurrence rate is high because it is not easy to reliably block the inflow artery of an AVM via an endoscopic approach [3].

An Interventional Radiology (IVR) method is efficient because it can achieve both diagnosis and treatment of AVMs. Embolization of an AVM requires complete embolization of the nidus without causing infarction of the organ [7]. If only the inflowing artery is embolized, development of collateral vessels is promoted, resulting in reestablishment of blood flow and reconstruction of a nidus [8, 9]. Therefore, recurrences also occur after TAE, resulting in it being considered a palliative form of treatment [10, 11]. There have been nine reports of intestinal AVMs treated with IVR in Japan. Although different embolic substances were used, all patients underwent TAE, and three of the nine (33%) subsequently had recurrences (Table 1) [3, 11–18]. There are some reports of TAE performed outside of Japan, but most of them are performed as neoadjuvant embolization, with TAE followed by surgery for radical cure [19, 20]. In addition, when embolic material passes through the nidus during arterial embolization, there is a risk of infarction occurring in other organs. In the present case, we considered that there would be a risk of hepatic ischemia if the embolic substance escaped into the blood vessels of the portal system, whereas transvenous nidus embolization after arrest of blood flow by a balloon might prevent unintentional embolism to, and ischemia of, other organs. The combined use of transvenous and transarterial embolization could achieve more complete eradication of the nidus and therefore prevent recurrence.

**Table 1:** Reports of intestinal AVMs treated with IVR in Japan

Author	Age	Sex	Symptom	Lesion site	Embolization method	Recurrence	Second-line treatment
Sekine et al. (2003) [12]	63	Female	Palpitation	Transverse colon	TAE (coils, spongel)		
Tuchikawa et al. (2003) [13]	62	Female	Membrane, Anemia	Right hemi colon	TAE (coils)	〇	IVR → Surgery
Yasui et al. (2007) [3]	80	Male	Membrane, Anal pain	Rectum	TAE (NBCA)	〇	Endoscopy Clipping → IVR → IVR
Okura et al. (2007) [14]	66	Male	Mambrane	Right hemi colon	TAE (coils)		
Yamada et al. (2010) [15]	75	Female	Membrane, Anemia	Transverse colon	TAE (NBCA)		
Fujisawa et al. (2012) [16]	64	Female	Membrane	Transverse colon	TAE (coils)	〇	IVR → Endoscopy Clipping → Surgery
Matsuura et al. (2014) [17]	68	Female	Membrane	Rectum	TAE (coils, NBCA)		
Komekami et al. (2017) [18]	38	Male	Membrane	Rectum	TAE (coils and gelatin particles)		
Ishikawa et al. (2020) [11]	86	Male	Membrane, Anemia	Rectum	TAE (microspheres)		
Our case	57	Male	Membrane	Rectum	Transvenous embolism + TAE (NBCA)		

The terms “arteriovenous malformations”, “intestinal tract”, “small intestine”, and “large intestine” were searched in the Central Medical Journal

In conclusion, we consider that embolization of a rectal AVM by a retrograde transvenous approach may achieve complete eradication of the nidus without the adverse event of unexpected embolism.
